# 1307. The Mycoplasma Conundrum

**DOI:** 10.1093/ofid/ofab466.1499

**Published:** 2021-12-04

**Authors:** Kenneth Rand

**Affiliations:** University of Florida, Gainesville, FL

## Abstract

**Background:**

Lockdown for Covid 19 between March 15 - 30, 2020 lead to sudden closures of schools, public gatherings, all but essential businesses, and stay-at-home orders. Between then and the end of April 2020, literally all enveloped respiratory viruses declined to virtually undetectable levels, suggesting a successful interruption of transmission. Weekly percentage positivity rates for M. pneumoniae and all other respiratory viruses from BioFire Syndromic Trends for weeks ending 3/7/2020- 4/24/2020.

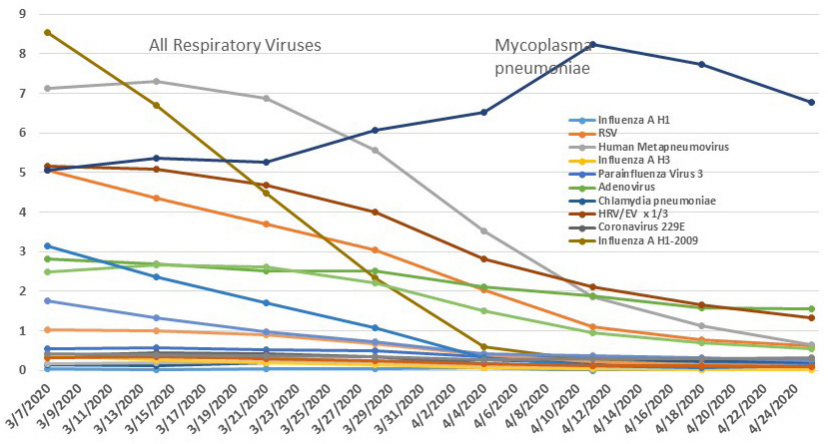

Weekly percentage positive rates are shown, with the Rhinovirus/Enterovirus rate divided by 3 and the M. pneumoniae rate multiplied by 10 to fit on the same scale.

**Methods:**

We used the percentage positivity rates from BioFire Syndromic Trends and from GenMark Diagnostics to examine the post lockdown response of M. pneumoniae versus other respiratory viruses on the Respiratory Virus Panel (RP 2.0)

**Results:**

As has been reported (Nawrocki J., et al, OFID 2021) and as shown in Figure 1, there was a rapid drop in the positivity rate for all enveloped respiratory viruses by 85.6% from an average rate of 2.014% positive for the week ending 3/14/20 to 0.29% for the week ending 4/18/20, while the positivity rate for M. pneumoniae actually increased by 44% from 0.536 % to 0.772%. The increase in M. pneumoniae positivity rate from its baseline of 0.51 ± 0.38 between 1/25/20 - 3/21/20 vs 0.71 ± 0.09 between 3/28/20 - 4/25/20 was significantly higher by t test, p=0.00574. Data from GenMark was available only monthly but also showed an upward rise from march to April, 2020.

**Conclusion:**

It is well documented that M. pneumoniae is transmitted through respiratory mechanisms, yet lockdown measures sufficient to dramatically reduce ordinary respiratory virus transmission had no comparable effect on transmission of Mycoplasma pneumoniae. It is also well known that M. pneumoniae persists in the respiratory tract as long as months after an infection. Therefore, it is possible that this reservoir continued to be a source of transmission for M. pneumoniae, even though lockdown measures effectively interrupted the enveloped respiratory viruses.

**Disclosures:**

**Kenneth Rand, M.D.**, **BioFire Diagnostics** (Advisor or Review Panel member, Research Grant or Support)

